# Psychosocial characteristics and HIV-related sexual behaviors among cisgender, transgender, and gender non-conforming MSM in China

**DOI:** 10.1186/s12888-021-03189-z

**Published:** 2021-04-17

**Authors:** Zhizhou Duan, Liyin Wang, Menglan Guo, Changmian Ding, Danqin Huang, Hong Yan, Amanda Wilson, Shiyue Li

**Affiliations:** 1 School of Health Sciences, Wuhan University, Wuhan, Hubei Province China; 2 The medical record department, The affiliated Dehong People’s Hospital of Kunming Medical University, Kunming, Yunnan Province China; 3 Institute for Infectious Disease Control and prevention, Hubei provincial Center for Disease Control and Prevention, Wuhan, Hubei Province China; 4 Division of Psychology, Faculty of Health and Life Sciences, De Montfort University, Leicester, UK

**Keywords:** Men who have sex with men, Cisgender, Transgender, Gender non-conforming, Psychosocial characteristics, HIV-related sexual behaviors

## Abstract

**Background:**

While a growing number of studies focus on men who have sex with men (MSM), they typically ignore the heterogeneity of gender minorities within the MSM population. The recognition of new sub-groups among gender minorities (i.e., transgender and gender non-conforming), who also identify as MSM, play a considerable role in new HIV infections in China. Information on the psychosocial factors and HIV-related sexual behaviors require further consideration to understand the prevalence of HIV infection among MSM within these gender minority sub-groups.

**Methods:**

From September 2017 to January 2018, MSM without HIV were recruited in Wuhan, Nanchang, and Changsha cities in China. Participants were asked to fill out a structured self-administered questionnaire to assess depression, perceived social support, resilience, identity concealment, and HIV-related risky sexual behaviors.

**Results:**

A total of 715 MSM completed the structured questionnaire, the number of MSM identifying as gender minorities were 63 and accounted for 8.8% of the population. Compared to the cisgender MSM population, transgender MSM were more likely to have a one-night stand/occasional partner (AOR = 3.49, 95% CI =1.02–11.98), to have sex after drug use in the past 6 months (AOR = 2.57, 95%CI =1.05–6.29), and to have reported a significantly lower likelihood of identity concealment (mean difference = − 3.30, 95%CI = -5.86, − 0.74, *P* = 0.01).

**Conclusions:**

The findings highlight the significance of providing targeted interventions for different gender minorities within the MSM population. Research is required to further understand the relationship between gender identity, mental health, and HIV-related sexual behaviors.

## Background

Gender minorities refer to those whose gender identity is not same as their birth-assigned sex (transgender) and those whose gender identity is not defined by the binary categories of women/men (gender non-conforming) [1, 2]. In recent studies around the world, there has been a call for further analysis of HIV interventions that target different gender identities among men who have sex with men (MSM) [3–5]. Previous research has shown that transgender and gender non-conforming MSM account for over 23% of the MSM population in Peru [6]. Jobson et al. found that 9% of individuals identified as gender minorities within the MSM population in a city of South Africa [7].

Individual MSM who identify as a gender minority report a higher likelihood of HIV risks in comparison with cisgender MSM [8, 9]. For example, compared to the cisgender MSM population, transgender MSM are at a higher risks of new HIV infections due to sociodemographic characteristics, having higher rates of substance use, and due to the higher engagement in sex work [10]. In a systematic review of MSM in low and middle income countries, transgender MSM had a higher prevalence of HIV and self-reported that they were more likely to engage in receptive anal intercourse without use of a condom [9, 11]. A recent survey in Shanghai also documented that transgender and gender non-conforming MSM bore a higher HIV burden, with the prevalence of HIV-infection reaching as high as 12.4% [12].

In addition, a previous study conducted in China has suggested that transgender and gender non-conforming populations self-report a higher incident of being bullied, higher experiences of neglect and abuse, and more depressive symptoms [13]. And the studies on transgender conducted in USA have found that psychosocial factors can increase the risk of HIV infection [14, 15]. Also, psychosocial factors including depression and stigma have been reported to increase the likelihood of HIV affecting in South African MSM [16]. Therefore, psychosocial characteristics of gender minorities can’t be ignored.

To date, the associated sub-groups within the MSM population, transgender and gender non-conforming, are not considered separate sub-groups within the MSM population in China [12]. Previous studies typically treat the MSM population as homogenous group and corresponding intervention measures have been generalized to target the MSM population as a whole [17], providing limited information on the impact of new HIV infections and transmission among MSM gender minorities [12, 18, 19]. In order to be effective in reducing HIV infections, growing evidence has suggested that it is important to create targeted intervention based on specific gender identity [7, 12].

Therefore, the present study aims to explore the relationship between gender identity, psychosocial factors, and HIV-related sexual behaviors to provide tailored interventions that can be more effective in reducing HIV infections in Chinese sexual minorities. First, this study estimated the prevalence of transgender and gender non-conforming individuals within the MSM population. Second, the study assessed the HIV-related sexual behaviors and psychosocial characteristics of each subgroup of gender minority MSM, in order to provide new evidence for targeted interventions among the different MSM gender minority populations.

## Methods

### Participants

Between September 2017 and January 2018, a baseline survey of a four-year cohort study was conducted in three provincial capitals of China: Wuhan, Nanchang, and Changsha. The cohort study aims to examine the effect mechanisms of perceived stigma on risk behaviors, psychology and HIV infection among Chinese MSM. Participants were recruited through several ways (i.e. routine HIV testing services, peer recommendation, and outreach activities), with the help of the local Centers for Disease Control and Prevention (CDC) in three cities, and MSM based organizations (Tongxing organization, Wuhan city; Xingyuan organization, Wuhan city and Qingcai organization, Changshai city, Nanchang City). The inclusion criteria for the study were: (1) they were 16 years or older; (2) they were men assigned at birth and had experience of having sex with men; (3) they were sexually active during the past 6 months; (4) they self-reported no HIV infection; (5) they provided written informed consent. The participants were asked to complete the electronic questionnaire on tablets (e.g. iPads) on their own in a quiet room in order to protect the individual privacy, and the trained students or volunteers were available to provide clarity if participants were unsure what the question was asking. All participants who finished the survey received 50 RMB (approximately US $7) as compensation for their time in this baseline survey. This study was approved by the Medical Ethics Committee of Wuhan University, China.

### Data collection

#### Socio-demographic characteristics

Socio-demographic information included: age, ethnicity (Han, others), current education level (high school or lower, college, graduate or higher), current employment (employed, unemployed), current marital status (unmarried, married, divorced), and monthly income (< 1000 Yuan, 1000–3000 Yuan, 3001–6000 Yuan, > 6000 Yuan). Sexual orientation was also measured by a single item question, “what’s your sexual orientation?”, and participants could choose from four options, “gay”, “bisexual”, “heterosexual”, and “unsure”.

#### Gender identity

Gender identity (the primary “independent” variable for the analysis) was measured by a single item, in order to identify transgender individuals, the question was asked “Do you agree with your sex assigned at birth?”, with three options included: “yes”, “no”, and “unsure”. The questions have been widely used both in China and other countries [7, 20]. In the study, we divided the MSM population into three categories according to gender identity, including cisgender MSM (those who agreed with their birth-assigned sex), transgender MSM (those who disagreed with their birth-assigned sex), and gender non-conforming MSM.

#### Psychosocial characteristics

The Center for Epidemiological Studies Depression (CES-D) was used to screen the symptoms of depression during the past week [21]. The CES-D has 20 items rated on a 4-point Likert scale ranging from “rarely or none of the time” (0) to “most or all of the time” (3). A higher total score indicates more depressive symptoms. The Chinese version of CES-D has been validated [22, 23] and the Cronbach’s alpha was 0.92 in this research.

The Multidimensional Scale of Perceived Social Support (MSPSS) was used to measure social support [24]. The scale consists of three subscales and each subscale includes four items. Response option for each item ranges from 1 (very strongly disagree) to 7 (very strongly agree). A higher total score indicates greater perceived social support. This scale have shown a good validity and reliability in the Chinese population [25]. The Cronbach’s alpha was 0.94 in this research.

The 10-item Connor-Davidson Resilience Scale (CD-RISC10) was used to measure resilience [26]. Responses were rated on a 5-point Likert scale ranging from “not true at all” (0) to “true nearly all the time” (4). A composite score was calculated, with higher total score indicating higher capacity of resilience. This scale has been confirmed validated in Chinese adults [27, 28] and the Cronbach’s alpha was 0.87 in this research.

Identity concealment was measured by a six-item, 5-point Likert subscale. This subscale was based on nondisclosure subscale developed by Testa [29] and further modified by Outland [30], which was suitable for LGBT population [30]. Items were summed to yield a composite scale score, with a higher score suggesting a higher likelihood of sexual orientation concealment. It has been confirmed validated among Chinese adults [31] and the Cronbach’s alpha was 0.91 in this study.

#### HIV-related sexual behaviors

Information on HIV-related sexual behaviors [32, 33] for the past 6 months was collected, including multiple sexual partners (2 or more were classified as having multiple sexual partners), male partner types (all were regular partners/acquaintances, all were one-night stand/occasional partner, or both regular partners/acquaintances and one-night stand/occasional partners), sex after drugs (yes or no), sex after drinking (yes or no), commercial sexual behavior (yes or no). Participants were also asked to answer frequency of condom use during anal sex in the past 6 months. The options included “never”, “sometimes”, “often”, “almost always” and “every time”. In data analysis, “never” “sometimes”, “often”, “almost always” were classified as inconsistent condom use.

### Statistical analysis

All analysis of data was conducted using SPSS19.0. The prevalence of gender minorities and 95% confidence interval (95%CI) were described. The Chi-squared tests and Fisher’s exact test were performed to explore the differences of socio-demographic characteristics and HIV-related risky behaviors by gender identity. In terms of psychosocial variables, multiple factor variance analysis was used to explore the relationships between different gender identity groups, which was adjusted for age, monthly income, and sexual orientation based on previous research [34–36]. The Bonferroni correction method was used to conduct a post-hoc analysis. Binary logistic analysis was used to further to explore how gender identity predicted HIV-related sexual behaviors including multiple sexual partners, sex after drugs, sex after drinking, commercial sexual behavior and inconsistent condom use, and the Hosmer-Lemeshow test was used to assess the binary logistic regression. While multinominal logistic analysis was used to assess the relationship between male partner types and gender identity, and Likelihood Ratio test, −2Log likelihood, and Pseudo R-square model fit were used to evaluate the model. In both binary logistic regression and multinomial logistic regression, gender identity was coded as independent variable, HIV-related risky sexual behaviors in the past 6 months were coded as dependent variables, and socio-demographic and psychosocial characteristics as covariates. The level of *P* < 0.05 (two-sides) was set for statistical significance in this study.

## Results

A total of 800 participants were invited into this survey, and 749 participants completed the questionnaire, indicating a response rate of 93.6% (Wuhan city, 93.8%; Changshai City, 91.5%; Nanchang City, 94.7%). Participants who did not have not sex with men in the past 6 months (*n* = 23), and who were not MSM (*n* = 11) were excluded. That left data for 715 MSM eligible for analysis. Of the eligible individuals, 502, 106, and 107 participants were recruited from Wuhan, Nanchang, and Changsha, respectively.

As shown in Table 1, the majority of participants were cisgender MSM (91.2%), transgender and gender non-conforming MSM accounted for 8.8% (95% CI: 6.7–10.9%) of the total MSM population. In all samples, 396(55.4%) MSM were between 16 to 25 years of age and 535(74.8%) had completed college or higher education. 604 (84.5%) of the participants were unmarried and 111(15.5%) were married or divorced. 663(92.7%) individuals were employed and 362(50.6%) described that their disposable income exceeded 3000 RMB monthly. 656(91.7%) reported that they were homosexual or bisexual, whereas 59(8.3%) reported as heterosexual or unsure.

**Table 1:** Socio-demographic characteristics by gender identity, n (%)

Variables	Total	Cisgender MSM	Gender non-conforming	Transgender	*χ*^*2*^	P
Total		652(91.2)	37(5.2)	26(3.6)		
Age group (in years) ^a^					–	0.95
16–25	396(55.4)	360(55.2)	22(59.5)	14(53.8)		
26–35	211(29.5)	193(29.6)	10(27.0)	8(30.8)		
36–45	69(9.7)	64(9.8)	2(5.4)	3(11.5)		
45–59	39(5.5)	35(5.4)	3(8.1)	1(3.8)		
Ethnicity ^a^					–	0.29
Han group	677(94.7)	618(94.8)	36(97.3)	23(88.5)		
Others	38(5.3)	34(5.2)	1(2.7)	3(11.5)		
Educational level					**6.54**	**0.04**
High school or lower	180(25.2)	156(23.9)	13(35.1)	11(42.3)		
College or higher	535(74.8)	496(76.1)	24(64.9)	15(57.7)		
Marital status					0.66	0.73
Unmarried	604(84.5)	553(84.8)	30(81.0)	21(80.8)		
Married/divorced	111(15.5)	99(15.2)	7(18.9)	5(19.2)		
Employment status ^a^					–	0.62
Employed	663(92.7)	605(92.8)	35(94.6)	23(88.5)		
Unemployed	52(7.3)	47(7.2)	2(5.4)	3(11.5)		
Monthly income (RMB)					10.18	0.12
< 1000	91(12.7)	80(12.3)	6(16.2)	5(19.2)		
1000–3000	262(36.6)	242(37.1)	10(27.0)	10(38.5)		
3001–6000	229(32.0)	215(33.0)	8(21.6)	6(23.1)		
> 6000	133(18.6)	115(17.6)	13(35.1)	5(19.2)		
Sexual orientation					**67.80**	**< 0.001**
Homosexual	520(72.7)	495(75.9)	11(29.7)	14(53.8)		
Bisexual	136(19.0)	116(17.8)	11(29.7)	9(34.6)		
Unsure/heterosexual	59(8.3)	41(6.3)	15(40.5)	3(11.5)		

^a^Fisher’s exact test; bolded value indicated *P* < 0.05

After adjusting for age, monthly income, and sexual orientation, gender identity was not associated with psychosocial characteristics, except for identity concealment (see Table 2). Specifically, cisgender MSM reported significantly higher scores of identity concealment than transgender (mean difference = − 3.19, 95%CI = -5.74, − 0.64, *P* = 0.01), while the differences among gender non-conforming MSM and the other two groups were not significant. It indicted that the cisgender MSM population were more likely to report high levels of identity concealment than transgender MSM.

**Table 2:** Psychosocial characteristics by gender identity, Mean ± SD (min-max)

Variables ^a^	Total	Cisgender	Gender non-conforming	Transgender	F	P
Depression	17.66 ± 10.50(0,58)	17.52 ± 10.46(0,58)	19.54 ± 11.27(2,48)	18.58 ± 10.43(2,44)	0.42	0.65
Perceived social support	60.42 ± 12.55(12,84)	60.57 ± 12.38(12,84)	60.46 ± 14.89(25,84)	56.46 ± 13.03(25,74)	1.14	0.32
Resilience	36.70 ± 8.48(10,50)	36.75 ± 8.51(10,50)	36.14 ± 8.45(10,50)	36.38 ± 8.11(17,50)	0.08	0.92
Identity concealment ^b^	17.05 ± 6.63(6,30)	17.19 ± 6.61(6,30)	16.68 ± 6.15(6,30)	14.00 ± 7.07(6,29)	**3.33**	**0.04**

*SD* standard deviation

^a^adjusted for age, monthly income and sexual orientation

^b^cisgender VS gender non-confirming: mean difference = − 0.52 (95%CI = -2.71, 1.68), *P* = 0.64; cisgender VS transgender: mean difference = −3.19 (95%CI = -5.74, − 0.64), *P =* 0.01 and gender non-confirming VS transgender: mean difference = −2.68 (95%CI = -0.64, 5.99), *P* = 0.11

Bolded value indicated *P* < 0.05

The characteristics of HIV-related behaviors were described in Table 3 and binary logistic regression and the multinominal logistic regression models showed good fit (see Table 4). Compared to cisgender MSM, transgender MSM were more likely to have a one-night stand/occasional partners (AOR = 3.49, 95% CI =1.02–11.98), and to have sex after drug use in the past 6 months (AOR = 2.57, 95% CI =1.05–6.29). No significant differences in HIV-related sexual risky behaviors were observed between gender non-conforming and cisgender MSM.

**Table 3:** HIV-related behaviors by gender identity, n (%)

Variables	Total	Cisgender MSM	Gender non-Conforming	Transgender	*χ*^*2*^	P
Multiple sexual partners					0.69	0.71
Yes	381(53.3)	306(46.9)	15(40.5)	13(50.0)		
No	334(46.7)	346(53.1)	22(59.5)	13(50.0)		
Male partner types ^a^					–	0.08
All were one -night stand/occasional partner	79(12.6)	68(11.9)	5(15.2)	6(28.6)		
All were regular partners/acquaintances	335(53.5)	313(54.7)	16(48.5)	6(28.6)		
Both regular partners/acquaintances and one-night stand/occasional partners	212(33.9)	191(33.4)	12(36.4)	9(42.9)		
Sex after drugs					**6.32**	**0.04**
Yes	100(14.0)	87(13.3)	5(13.5)	8(30.8)		
No	615(86.0)	565(86.7)	32(86.5)	18(69.2)		
Sex after drinking					0.85	0.65
Yes	281(39.3)	258(39.6)	12(32.4)	11(42.3)		
No	434(60.7)	394(60.4)	30(81.0)	21(80.8)		
Commercial sexual behavior ^a^					**–**	**0.049**
Yes	12(1.9)	9(1.6)	1(3.0)	2(9.5)		
No	614(98.1)	563(98.4)	32(97.0)	19(90.5)		
Inconsistent condom use					1.81	0.41
Yes	286(49.2)	257(48.4)	18(60.0)	11(55.0)		
No	295(50.8)	274(51.6)	12(40.0)	9(45.0)		

^a^Fisher’s exact test; bolded value indicated *P* < 0.05

**Table 4:** The relationship between gender identity and HIV-related sexual behaviors

Variables ^a b c^	Gender non-conforming				Transgender			
	B	P	AOR	95% CI	B	P	AOR	95% CI
Multiple sexual partners (yes)	−0.13	0.73	0.88	0.43–1.80	0.12	0.77	1.13	0.51–2.52
Male partner type								
All were regular partners/acquaintances	ref				ref			
All were one-night stand/occasional partners	0.16	0.78	1.17	0.38–3.64	**1.25**	**0.047**	**3.49**	**1.02–11.98**
Both regular partners/acquaintances and one-night stand/occasional partners	0.12	0.78	1.12	0.49–2.56	0.73	0.18	2.08	0.71–6.09
Sex after drugs (yes)	−0.17	0.75	0.84	0.30–2.39	**0.94**	**0.04**	**2.57**	**1.05–6.29**
Sex after drinking (yes)	−0.49	0.20	0.61	0.29–1.30	−0.04	0.93	0.96	0.43–2.18
Commercial sexual behavior (yes)	0.55	0.65	1.73	0.17–18.00	1.71	0.06	5.51	0.91–33.39
Inconsistent condom use (yes)	0.58	0.16	1.79	0.79–4.08	0.08	0.86	1.09	0.43–2.78

*AOR* adjust odds ratio, *CI* confidence interval; bolded value indicated *P* < 0.05

^a^All models controlled for age, ethnicity, educational level, marital status, employment status, monthly income, sexual orientation, depression, perceived social support, resilience and identity concealment

^b^Hosmer and Lemeshow Test: multiple sexual partners, Chi-square = 7.69, df = 8, *P* = 0.47; sex after drug, Chi-square = 7.26, df = 8, *P* = 0.51; sex after drinking, Chi-square = 4.85, df = 8, *P* = 0.77; commercial sexual behaviors, Chi-square = 2.24, df = 8, *P* = 0.97; inconsistent condom use, Chi-square = 2.52, df = 8, *P* = 0.96. Model fit of male partner types: -2Log likelihood =1156.90, Chi-square = 51.378, df = 26, *p* = 0.002; Cox and Snell square = 0.08, Nagelkerke square = 0.09, McFadden = 0.04

^c^gender identity was independent variable and Reference Category were cisgender MSM

## Discussion

To the best of our knowledge, this is the first study to explore gender identity differences in psychosocial characteristics and HIV-related sexual behaviors among the MSM subgroup populations in China. In the current study, compared to cisgender MSM, those in the transgender subgroup were more likely to engage in a one-night stand/occasional partners, have sex after drug use, and reported a lower likelihood of identity concealment. Consistent with Jobson’s research, gender identity played an important role in HIV transmission among the MSM population [7]. Hence, the findings from this study suggest that effective HIV interventions within the MSM population should be separated by the different gender identities.

The study showed that the proportion of transgender and gender non-conforming accounted for 8.8% of the total MSM population. A study conducted in Peru reported that 23% of sample were identified as gender minorities among the MSM population [6], while another study found that 7.2% of the MSM population identified as transgender women [32]. This existing discrepancy could be attributed to various assessment tools of gender identify, different cultural norms, or study settings. Though our sample is limited to the recruitment from the central part of China, this study helps to establish the initial epidemic profile of risk factors for transgender and gender non-conforming MSM in China.

Non-significant associations between depression, resilience, perceived social support, and gender identity in the MSM population was consistent with Sandfort’s research [37]. The cisgender MSM population were more likely to report high levels of identity concealment than transgender MSM. The lower scores of identity concealment among transgender identifying MSM could mean that transgender MSM have to tolerate more discrimination when compared with the cisgender MSM population [38], which may increase the frequency of condomless anal sex [39] or other HIV-related risk behaviors (e.g. male partner type) [40] and in turn increase their risks of HIV infection. Thus, future research should further explore the relationship between gender identity and psychosocial characteristics within the MSM sub-populations, using additional measures such as discrimination.

Some studies conducted in community population showed that transgender individuals were more likely to have a one-night stand/occasional partners and sex after drug use, when compared to the cisgender MSM population [12, 41]. A study in Brazil reported that transgender individuals engaged in more HIV-related sexual behaviors than MSM individuals, including having a higher number of sexual partners, being more likely to engage in sex [42]. Similarly, our study conducted among the MSM population found that compared with cisgender MSM, transgender MSM were more likely to have sex after drug use and have a one-night stand/occasional partners. This is possibly because that transgender MSM have a dual minority identity being both transgender and MSM [43] and due to this dual identity, they perceive stigma when seeking support from service, while the cisgender MSM population do not. The dual minority identity of transgender MSM, as stated, are shown to have a higher level of discrimination and engage in more risky sexual behaviors [7]. Consequently, these results suggested that we should focus on gender minority sub-groups, as transgender MSM have different risks than the larger groups of cisgender MSM. Considering the high likelihood of one-night stand/occasional partners and sex after drug use among transgender MSM, it is crucial to increasing the awareness of HIV infection risk about above behaviors for this sub-group population, in turn reducing the frequency of HIV-related sexual behaviors. In addition, government should strengthen drug administration in order to reducing the behavior of sex after drug use.

For gender non-conforming MSM, they may or may not consider themselves to be transgender [44, 45], so they face unique life experiences and may have different psychosocial health outcomes [45]. Halley’s research [45] also showed gender non-conforming MSM populations were more likely to have poor mental health and distress in comparison with transgender individuals. However, in this study, gender non-conforming MSM were not found to have significant differences in psychosocial characteristics and HIV-related sexual behaviors in comparison with the cisgender MSM. This is possibly due to small sample size in the study. Therefore, future research needs to increase sample size for further focusing on the population.

Several limitations should be noted in this current study. First, participants were recruited in three Chinese central cities by convenient sampling, and the sample size of transgender MSM and gender non-conforming MSM was small, which impacted the generalizability of the findings. Second, psychosocial outcomes were measured by four self-reported scales, not considering other variables like anxiety, self-esteem, self-efficacy, and copying styles. Also, other HIV-related behaviors (PrEP/PEP, substance use) weren’t included in the study, and detail information about types of drug or drinking during sex, frequency, duration of use wasn’t obtained. It is necessary that additional psychosocial and behavioral outcomes are incorporated in further research exploring the relationship between gender identity, psychosocial charactristics, and HIV-related risk behaviors. Third, there was a potential for social desirability bias in the psychosocial characteristic assessment. Fourth, the study ignored the differences within the transgender community, further research is needed to understand the difference between transgender women and transgender men in China. Despite these limitations, this study highlights the prevalence of transgender and gender non-conforming MSM in China, and found gender identity differences in psychosocial characteristics and HIV-related sexual behaviors. There is a need for further studies focusing on the association between gender minorities, psychosocial characteristics and HIV related risk behaviors with larger sample and considering more factors.

## Conclusions

In conclusion, the proportion of transgender and non-conforming MSM was 8.8% in the current study. Compared with the cisgender MSM population, transgender MSM reported low levels of identity concealment, but were more likely to engage in HIV-related sexual behaviors, including having a one-night stand/occasional partners and sex after drug use. It is crucial to provide targeted interventions for transgender MSM, a high-risk subgroup. Further research is also needed to focus on the mechanism of the relationships between gender identity, psychosocial characteristics, and HIV-related risky sexual behavior among the MSM sub-groups for effectively reducing HIV infection risk.

## Data Availability

Data is available from the corresponding author upon reasonable request.

## Abbreviations

MSM: Men who have sex with men
HIV: Human immunodeficiency virus
CDC: Centers for Disease Control and Prevention
CI: Confidence interval
AOR: Adjust odds ratio
SD: Standard deviation
AIC: Akaike Information Criterion
BIC: Bayesian Information Criterion
